# Novel flexible and conformable composite neutron scintillator based on fully enriched lithium tetraborate

**DOI:** 10.1038/s41598-023-31675-9

**Published:** 2023-03-23

**Authors:** Felix Pino, Jessica Carolina Delgado, Sara Maria Carturan, Giorgia Mantovani, Matteo Polo, Daniela Fabris, Gianluigi Maggioni, Alberto Quaranta, Sandra Moretto

**Affiliations:** 1 Department of Physics and Astronomy “Galileo Galilei”, University of Padova, Padua, Italy; 2 Laboratori Nazionali di Legnaro, INFN, Legnaro, Italy; 3 Department of Physics and Earth Science, University of Ferrara, Ferrara, Italy; 4 Department of Industrial Engineering, University of Trento, Povo, Italy; 5 Padova Section, INFN, Padua, Italy; 6 TIFPA-Trento Institute for Fundamental Physics and Applications, INFN, Povo, Italy

**Keywords:** Experimental nuclear physics, Experimental particle physics

## Abstract

Thermal neutron detection is a key subject for nuclear physics research and also in a wide variety of applications from homeland security to nuclear medicine. In this work, it is proposed a novel flexible and conformable composite thermal neutron scintillator based on a fully enriched Lithium Tetraborate preparation ($^{6}$Li$_2^{10}$B$_4$O$_7$) combined with a phosphorescent inorganic scintillator powder (ZnS:Ag), and is then distributed into a polydimethylsiloxane matrix. The proposed scintillator shows a good neutron detection efficiency (max. $\sim$ 57% with respect to the commercial EJ-420), an average light output of $\sim$ 9000 ph/neutron-capture, a remarkable insensitivity to $\gamma$-rays (Gamma Rejection Ratio <10$^{-11}$), and an extraordinary flexibility, so as to reach extremely small curvature radii, down to 1.5 mm, with no signs of cracking or tearing. Its characteristics make it suitable to be employed in scenarios where non-standard geometries are needed, for example, to optimize the detector performance and/or maximize the detection efficiency. Finally, the response of a hybrid detector made of a plastic scintillator, wrapped with the proposed scintillator, coupled to a silicon photomultiplier array is described, and the excellent discrimination between $\gamma$-rays, fast and thermal neutrons resulting from data processing is demonstrated.

## Introduction

Nowadays, neutron detection is a subject of steadily growing interest in nuclear and particle physics research, but also it is fundamental in a wide variety of applications such as homeland security^1–3^, neutron monitoring (in power plants and radioactive waste repositories^4–6^), material analysis^7^, hydrology^8^, radiation protection^9^, industrial procedures, nuclear medicine^10^, etc. Presently, the increasing interest in the development of new nuclear power plants, pushed forward by the current, dramatic energy crisis, demands modern safeguards equipment to ensure that nuclear material is not diverted from peaceful uses. Nuclear medicine applications, such as the boron neutron capture therapy (BNCT), are current cutting-edge therapeutic facilities in which reliable neutron sensors are needed. In the BNCT specific case, required responsively is toward thermal neutrons, whose capture by boron-10 doped cancer cells generates ionizing radiations able to destroy the sick surrounding tissue^10^. Another example where the usage of neutrons sensors is greatly recommended, is the proton therapy; in this case, secondary radiations are produced inside the body while the high-energy primary proton beam is passing through the human tissue, thus causing the generation of a mixed radiation field, including thermal neutrons. Also, in that case, the use of a phantom to predict with accuracy the type and entity of secondary radiation is of paramount importance to describe in detail the effects of radiation-body interaction, thereby avoiding radiation-induced damage to healthy tissues surrounding the tumor cells^11^.

Thermal neutron detection with negligible (or recognizable) response to $\gamma$-rays is of great interest, and different scintillation materials have been developed. From the ones that have a negligible response to the gamma-rays, there are the scintillator screens, which consist of a mixture of compounds (usually a neutron converter such as $^6$Li or $^{10}$B compounds mixed with inorganic scintillators such as: ZnS:Ag^12^, ZnS:Cu^13^, GYAGG^14^, perovskite nanocrystals^15^, etc.) spread over a substrate (aluminium, polymethyl methacrylate). These detectors are commonly used for neutron imaging and neutron radiography and for radiation portal^1,16,17^.

On the other hand, simultaneous real-time discrimination of $\gamma$-rays and neutrons is of interest for several homeland security applications, and for nuclear safety and waste management operations^4,18,19^. In this respect, there are recent scintillation compounds (e.g. Gd$_2$O$_2$S:Tb$^{3+}$^20^, CLYC^21^, CLLB^22^) in which the neutron converter is included in the scintillator composition. Some of them exhibit high neutron detection efficiency, but, usually they are rigid and fragile. These type of detectors are commonly used on homeland security applications, for special nuclear material identifications, nuclear experiments, etc.

In previous research done by our group, a valid alternative has been found. It is constituted by a thin sheet produced by mixing ZnS activated with Ag (ZnS:Ag powder) with $^{6}$LiF nano-crystals dispersed in a polysiloxane rubber. The detector showed an optimum performance, providing a high thermal neutron detection efficiency^23^, as compared to the EJ-420 commercial scintillator.

**Figure 1:**
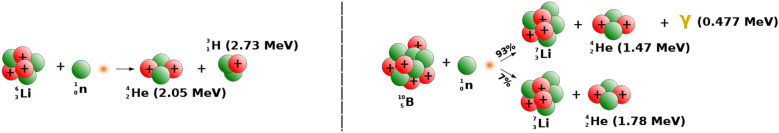
Thermal neutron capture reaction scheme of the $^{6}_{3}$Li and $^{10}_{5}$B.

In this work, we synthesized fully enriched Lithium Tetraborate ($^{6}$Li$_2^{10}$B$_4$O$_7$) and then entrapped the nanocrystals in flexible siloxane support loaded with an inorganic scintillator (ZnS:Ag powder). The use of $^{6}$Li$_2^{10}$B$_4$O$_7$ (LiBO) is expected to enhance the probability to capture thermal neutrons, due to the double neutron capture reaction pathway (see Fig. 1). The light reaction products (alpha and tritium) emitted after the neutron capture reactions might escape the LiBO grain, travel through the matrix and hit the scintillating grain (ZnS:Ag). Then, the scintillation light produced is transmitted through the silicone until reaching the read-out device (photomultiplier, SiPM, etc) window. It is worth to mention that generally only a portion of these scintillation photons is transmitted, because of the self-shielding effect due to the opacity of the material.

Several synthesis routes can be pursued to achieve LiBO nanoparticles: the “Pechini” method has been applied by Khalilzadeh to control the size of the growing crystal during precipitation, using polyvinylpyrrolidone as structure-directing agent^24^, and very recently this approach has been followed by Frangville^25^ to produce LiBO loaded plastic scintillator based on polystyrene as a matrix and doped with an extra amount of primary dye to enable the discrimination of fast neutrons and $\gamma$-rays.

In this work, we propose a direct thermal synthesis for the achievement of LiBO nanocrystals with optimal yield and crystal quality. The nanopowders are mixed with the inorganic scintillator ZnS:Ag (commercial name EJ-600 from Eljen Technology, grain size is claimed to be 8 $\upmu m$) and entrapped into polydimethylsiloxane as a scaffold, thus producing a lightweight, flexible and thin thermal neutron sensing material. The main goal of this work is to develop a novel flexible and conformable composite scintillator to be used as a highly efficient thermal neutron detector.

By other hand, simultaneous detection of thermal neutrons, fast neutrons, and $\gamma$-rays with a single device is a challenging task, that has been addressed by applying particle discrimination techniques (based on analog and digital pulse shape analysis of the signals). The possibility to achieve triple particle discrimination has been explored using different materials: organics, plastics and liquids doped with suitable fluorophores and soluble $^{10}$B or $^{6}$Li compounds, and “phoswich” configurations, in which a thermal neutron scintillation detector, often loaded with a $^{6}$Li compound, is optically coupled with an organic scintillator, sensitive to $\gamma$-rays and fast neutrons. Depending on the scope to be fulfilled, the environment of usage, and the desired accuracy, each of these methods has advantages and disadvantages. Some relevant results are described in Refs.^26–34^.

In order to highlight the potentialities of the new proposed thermal neutron scintillator, a hybrid detector configuration was studied, i.e. commercial plastic scintillators (EJ-299 and EJ-276G from Eljen Technology) were wrapped with the LiBO-based scintillator. In terms of practical applications, it means that $\gamma$-rays, fast and thermal neutrons can be detected and discriminated simultaneously. This configuration is easy and fast to assemble, resistant to bending without tearing or cracking, and it is thermally resistant, PDMS (polydimethylsiloxane) with a thermal decomposition temperature of T$_{d}$ = 310$^{\circ }$C. Furthermore, due to the flexibility of the LiBO-based scintillator, this detector can be implemented in a wide range of applications, particularly in difficult geometrical conditions, such as in nuclear fuel cycle facilities. In such cases, the device can be easily bent to cover a small/large curved surface, resulting in a higher thermal neutron detection efficiency.

The possibility to achieve a fully flexible hybrid device composed of a conformable light-emitting sensor coupled with a thin, pliable photoconverter is extremely exciting. This possibility is close at hand, taking into account the cutting-edge technology that very recently led to ultra-highly sensitive organic phototransistors (OPTs), with an unprecedented limit of detection as low as few nW/cm$^2$^35^. Therefore, the experimental work and the herein reported measurements under thermal neutrons irradiation have been pursued to assess the key features of this thin, nanocomposite, light emitting material, thereby providing a proof-of-principle for the development of a fully flexible thermal neutrons detector, composed of an elastic sensor coupled to bendable organics-based transistors.

## Results

### Synthesis of the fully enriched lithium tetraborate powder

**Figure 2:**
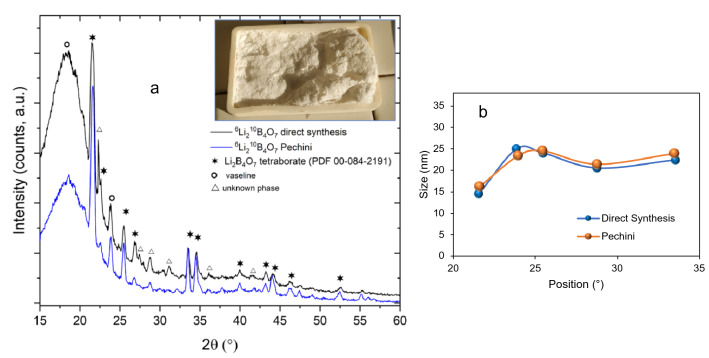
(**a**) XRD pattern of LiBO prepared by direct synthesis or Pechini method, showing the Li$_2$B$_4$O$_7$ phase assignment. The photo in the graph inset shows the powder extracted from the muffle furnace. (**b**) Crystallite size as derived from the Scherer formula is reported for both syntheses.

The LiBO powders obtained using either the Pechini method or the direct synthesis, as described in the experimental section, have been analyzed by High-Resolution X-ray Diffraction (HR-XRD). The diffraction pattern is shown in Fig. 2. In the case of Pechini synthesis, the peaks are assigned to an almost pure lithium tetraborate phase, as indexed in the graph (Li$_2$B$_4$O$_7$ tetraborate, PDF 00-084-2191), and it is in agreement with previous literature^24,25^. Other phases are present, as evidenced in the graph, but assignment to a precise structure is difficult owing to the superposition of peaks with tetraborate.

**Figure 3:**
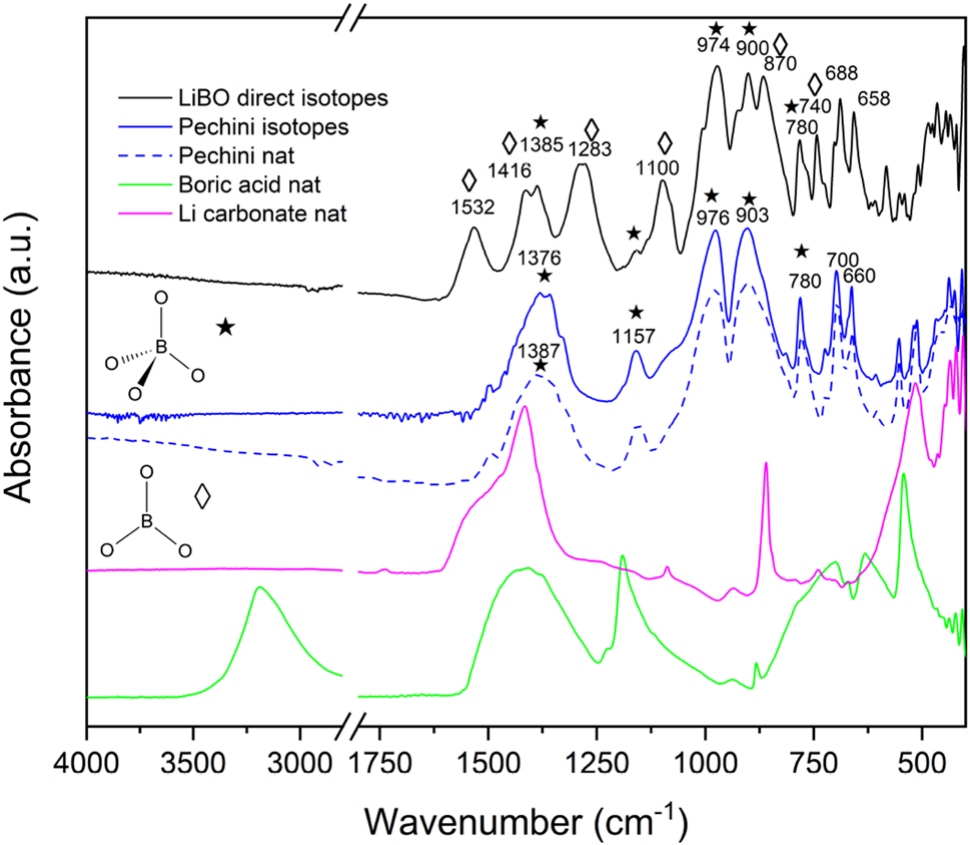
Infrared spectra collected in ATR mode of Lithium tetraborate obtained by direct synthesis or Pechini (Khalil) method. The spectra of precursors are also reported for comparison.

On the other hand, the direct thermal synthesis leads to a mixture of tetraborate and another phase, not easy to identify. There are strong correlations with the findings of Betourne^36^, which point to the presence of triborate compounds (LiB$_{3}$O$_{5}$). This finding is supported by the results of infrared spectroscopy, shown in Fig. 3, where the spectra of powders are collected in Attenuated Total Reflectance mode (FT-IR, ATR). The presence of $^{10}$B in the network instead of natural boron can induce moderate shifts in the peak position, as evidenced in literature^37,38^. In our spectra, taking into account the isotopic effect, the successful synthesis of Lithium borate is confirmed, although in the case of direct synthesis a clear, dominant presence of trigonal coordination in the boron compound is pointed out by the intense peaks in the region above 1200 cm$^{-1}$. On the other hand, following the Pechini synthesis, a major contribution comes from tetragonal coordination, as demonstrated by signals in the range 850–1100 cm$^{-1}$.

### Preparation of the LiBO/ZnS:Ag-based scintillator

The Lithium Tetraborate preparation is mixed with a fraction of the ZnS:Ag scintillator (EJ-600 powder from Eljen Technology, Texas, USA), and then all this is dispersed in a PDMS matrix. Six samples were made, each with a different percentage of ZnS:Ag/LiBO mix (20%v/v, 30%v/v, and 40%v/v) and PDMS matrix, as well as a different weight ratio between the ZnS:Ag powder and the LiBO preparation (3:1 and 2:1, respectively). The weight ratios have been chosen in analogy with commercial scintillators. Taking into account that all the thermal neutron measurements of the proposed scintillator have been benchmarked against the EJ-420 commercial scintillator, similar formulations have been synthesized to get a more direct comparison.

**Figure 4:**
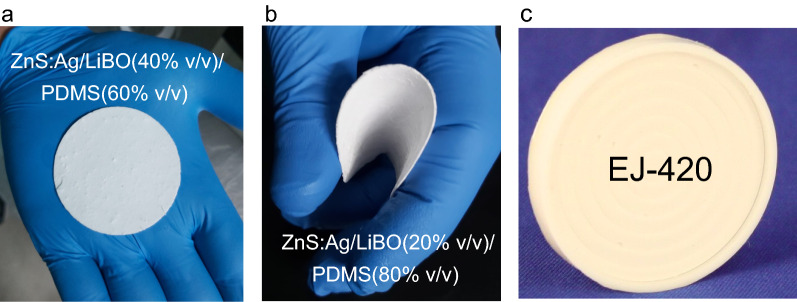
Pictures of two samples of the LiBO/ZnS:Ag-based scintillators produced (**a**) ZnS:Ag/LiBO (3:1) (40% v/v)/PDMS and (**b**) ZnS:Ag/LiBO (3:1) (20% v/v)/PDMS. (**c**) Commercial EJ-420 thermal neutron detector.

In Fig. 4a,b, two of the LiBO/ZnS:Ag-based scintillators produced are shown, all of them with the same physical characteristics: white color, excellent flexibility, a diameter of 50 mm and thickness of $\sim$ 0.4 mm. In Fig. 4c the commercial analog detector EJ-420 ($2^{\prime \prime}$ dia. $\times 2^{\prime \prime}$ thick.), from Eljen Technologies Texas-USA, is shown for comparison purposes. As can be seen, the EJ-420 is supported and surrounded by a rigid plastic material.

### Flexibility of the LiBO/ZnS:Ag-based scintillator

**Figure 5:**
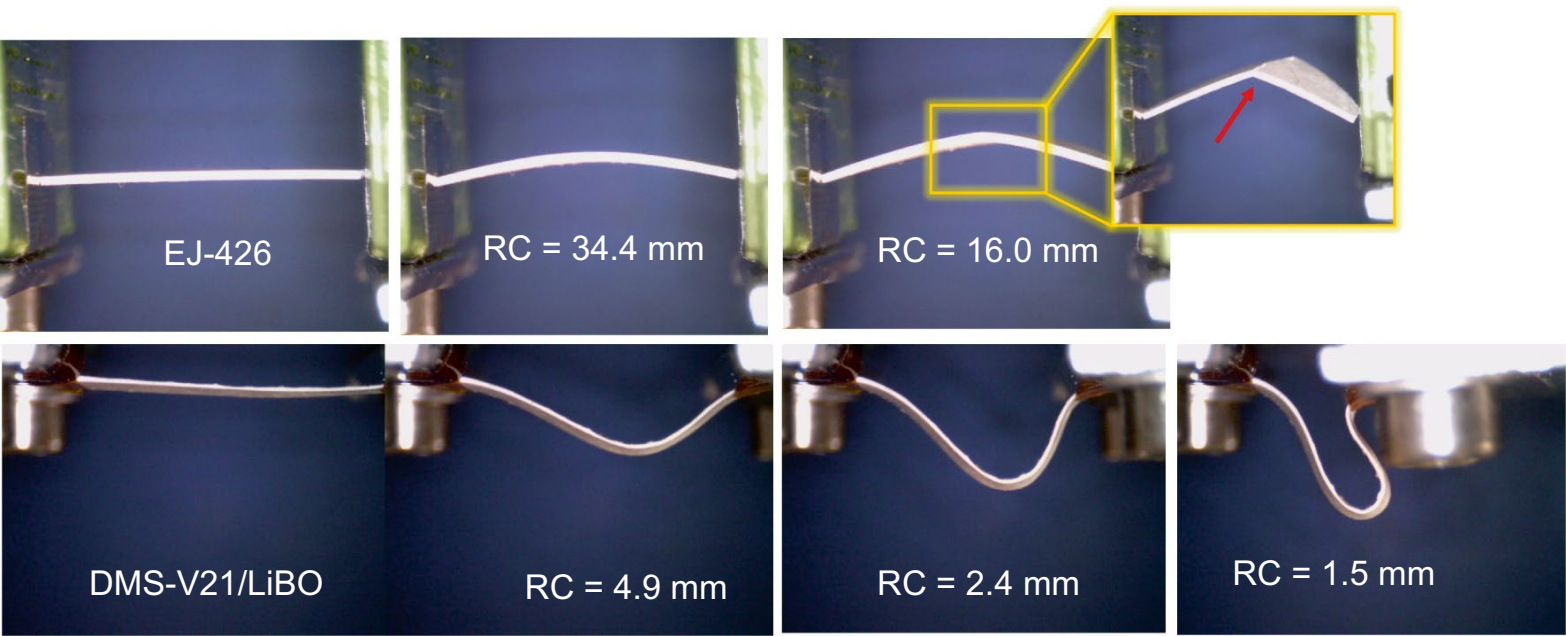
Pictures of the commercial scintillator EJ-426 (top row) and the ZnS:Ag/LiBO/PDMS detector (bottom row) clamped in a vice and bent at decreasing curvature radii.

A flexibility test was performed on the LiBO-based scintillator. For comparison purposes, the same test was executed on the EJ-426 detector, from Eljen Technologies Texas-USA. The EJ-426 thermal neutron detector has the same composition as the EJ-420, but the material is dispersed in a colorless binder, producing a thin composite layer of about 0.25 mm displaying minimal flexibility. The test consists of holding a scintillator piece from two edges, then approaching these points over the line that connects them in order to bend the material at decreasing radii, with no restrictions on the bending radius, as shown in Fig. 5. As can be observed the ZnS:Ag/LiBO/PDMS scintillator can easily reach a small curvature radius, demonstrating exceptional flexibility. The siloxane-based blend that composes the scintillator does not reach the rupture point. The elastomeric nature of the matrix and the intimate powder mixing allow the achievement of a composite with withstand extremely small curvature radii without failure. Flexibility is tested down to a bending radius of 1.5 mm as shown in Fig. 5. In contrast, the EJ-426 detector cannot withstand a curvature radius lower than 16 mm.

### Thermal neutron response of the LiBO/ZnS:Ag-based scintillators

Six different samples of ZnS:Ag/LiBO/PDMS scintillators were tested using the neutron flux generated by the $^{7}$Li(p,n)$^{7}$Be reaction (proton energy $\sim$ 5.5 MeV) at the Van de Graaff accelerator at the Laboratori Nazionali di Legnaro—INFN. The mono-energetic fast neutrons ($\sim$ 3.8 MeV at 0$^{\circ }$) were moderated by surrounding the detector assembly with a few centimeters of polyethylene (in Ref.^29^ it has been demonstrated that $\sim$ 6 cm is a reasonable thickness).

**Figure 6:**
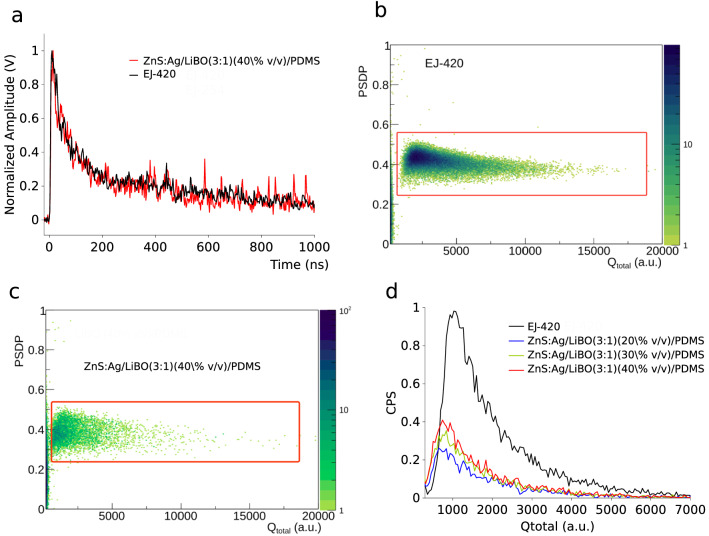
(**a**) Typical waveform of the detector tested. 2D-PSD plots corresponding to the measurements with the neutron flux at the CN accelerator with (**b**) the EJ-420 detector and (**c**) the ZnS:Ag/LiBO (3:1) (40% v/v)/PDMS detector. (**d**) Energy spectrum of the detector tested with the $^{252}$Cf source.

In Fig. 6a typical waveforms corresponding to thermal neutron-induced events of the tested detectors are shown. The decay time of the ZnS:Ag/LiBO-based scintillator signal is $\sim$ 200 ns, which is in agreement with the decay time exhibited by the EJ-420 signals. Taking into account that the fluorophore entrapped into the light emitting composite sensor is the same (ZnS:Ag) used in EJ-420, a negligible difference between the shape of the signals is expected. The analysis of the signals is performed by following the so-called double integration method or charge comparison method^12^, in order to separate the thermal neutron induced events from other kinds of events, such as: $\gamma$-rays induced events, noise, etc. The short integration, $Q_{short}$, includes the fast rise time region and part of the decay component of the signal, whereas the total integration, $Q_{total}$, covers the whole signal. The Pulse Shape Discrimination (PSD) parameter is obtained as follows:

1 $$\begin{aligned} PSD=\dfrac{Q_{total}-Q_{short}}{Q_{total}}=\dfrac{Q_{tail}}{Q_{total}}, \end{aligned}$$

where $Q_{tail}$ can be interpreted as the integral of the tail of the signal. A 2D-PSD plot consisted in plotting the PSD parameter of each event as a function of its $Q_{total}$. In Fig. 6b,c the 2D-PSD plots of the EJ-420 and one of the ZnS:Ag/LiBO-based scintillator are shown respectively. The long (G$_L$) and short (G$_S$) integration gates of the signals were optimized until the best visual distribution and separation were found as related to thermal neutron events in the 2D-PSD plots. The best performing values were found to be G$_L$ = 1.7 $\upmu$s and G$_S$ = 0.6 $\upmu$s for all the detectors.

Given that the ratio of the number of $^{10}$B and $^{6}$Li nuclei in the LiBO is 2:1, and that the (n$_{th}$,$\alpha$) reaction cross-section for the $^{10}$B is approximately four times higher, it is reasonable to assume that the majority of the thermal neutron events recorded are due to capture reaction on $^{10}$B. Nevertheless, the signals induced by neutron capture on the $^{10}$B are expected to have smaller amplitudes (and also smaller integrals) than those induced by neutron captures on $^{6}$Li, because the signal in the case of $^{10}$B is primarily made up of alpha particle induced scintillation ($\sim$1.5 MeV), whereas the scintillation signal induced by $^{7}$Li is highly quenched. Specifically, from SEM images (see Supplementary Fig. S1), the dimension of the LiBO grains (average of $\sim$ 2 $\upmu$m) was found to be very close to the range of $^7$Li ions (E = 0.84 MeV) in LiBO (density = 2.4 g/cm$^3$), which is 2.1 $\upmu$m. So, the probability that a $^7$Li ion escapes from a LiBO grain is expected to be low, furthermore, if it escapes, the energy deposited on a ZnS:Ag grain (and subsequently the light yield) would be low in comparison with the one deposited by the alpha particle. Figure 8 illustrates the path of the daughter products on the polydimethylsiloxane matrix and on a LiBO target, results obtained using the Transmission of Ions in Matter (TRIM)^39^ simulation. As a matter of fact, the quenching effect in scintillators is proportional to the specific energy loss, dE/dx, of the particle, and as Li ions are heavier and have larger Z with respect to the alpha particles, they have higher dE/dx, hence higher quenching effect is expected for Li ions^40,41^. Conversely, in the case of capture on $^6$Li, both reaction products, i.e. alpha ($\sim$ 2 MeV) and tritium ($\sim$ 2.7 MeV), contribute to the signal generation. It can be seen in Fig. 6d that the $Q_{total}$ mean values of the ZnS:Ag/LiBO-based scintillator spectra ($\sim$ 1200) are smaller than the one displayed by the EJ-420 ($\sim$ 2000).

**Figure 7:**
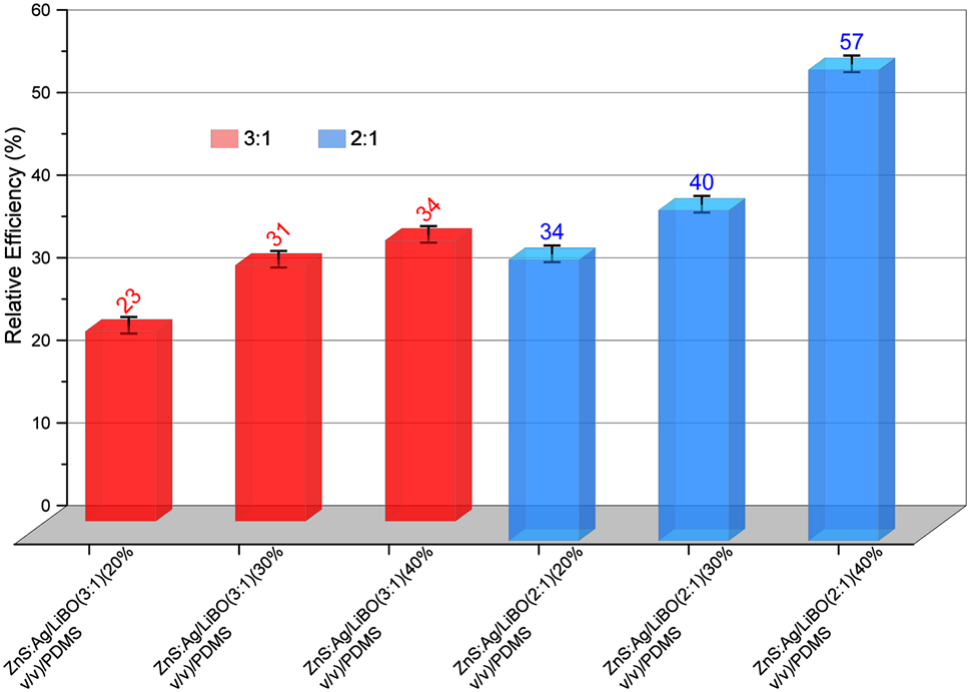
Thermal neutron relative efficiencies of the ZnS:Ag/LiBO-based detectors with respect to the EJ-420 detector.

The detection efficiency of a neutron detector is a crucial parameter and it should be evaluated with accuracy. However, an accurate measurement of the absolute detection efficiency of a neutron detector is a difficult task. One of the main obstacles is having access to a very well characterized neutron field. It means that the neutron emission rate (intensity) and the angular distribution as a function of the neutron energy should be known. Therefore, as it is not possible at the moment to access a neutron facility with full and accurate description of the irradiation field, the neutron detection efficiency is reported with respect to the EJ-420 (commercial detector) performance, which, according to its producer, exhibits an absolute thermal neutron detection efficiency of 55%. The thermal neutron counting rate of each detector has been determined by integrating the light output ($Q_{total}$) spectrum (see Fig. 6d). In Fig. 7 it is reported the thermal neutron detector efficiency relative to the EJ-420 detector for each tested scintillator. The uncertainties calculated in the results follow the standard procedures of error propagation. In terms of detection efficiency, samples with a weight ratio of 2:1 between ZnS:Ag powder and LiBO preparation show the best responses; in fact, the best result is obtained with the ZnS:Ag/LiBO (2:1) (40% v/v)/PDMS scintillator, which has a thermal neutron detection efficiency of $\sim$ 57% when compared to EJ-420. As reported in^16^, the typical thermal neutron detection efficiency of ZnS-series phosphors with neutron converters (including commercial ones) ranges between 25 and 40%. The proposed LiBO-based scintillator (with an estimated efficiency between 13 and 31%) offers a response as good as other similar scintillation detectors, demonstrating that it is sufficient for most detection applications. It is worth to observe from data in Fig. 7 that the efficiency steadily increases with LiBO concentration, thus indicating that an even higher value can be achieved with a further loading of thermal neutron sensitive nuclei. This encouraging outcome and the outstanding mechanical properties above demonstrated make the ZnS:Ag/LiBO-based scintillator an optimal choice as thermal neutron detector in specific challenging applications.

**Figure 8:**
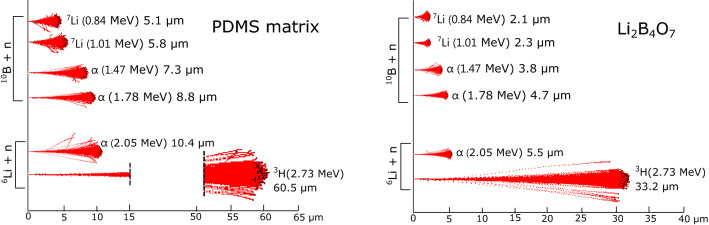
Simulated paths range on the polydimethylsiloxane matrix and on a LiBO target of the daughter products coming from the $^{6}$Li and $^{10}$B thermal neutron reaction. The simulation has been performed using the code Transmission of Ions in Matter (TRIM)^39^.

According to the manufacturer, the Lithium content of the EJ-420 is 9.03 $\times$ 10$^{20}$ $^{6}$Li atoms/cm$^{2}$ ($\sim$ 9 mg/cm$^{2}$). Our ZnS:Ag/LiBO-based scintillator, with 40% v/v of ZnS:Ag/LiBO (2:1), contains 1.34 $\times$ 10$^{20}$ $^{6}$Li atoms/cm$^{2}$ ($\sim$ 1.3 mg/cm$^{2}$) and 2.67 $\times$ 10$^{20}$ $^{10}$B atoms/cm$^{2}$ ($\sim$ 4.4 mg/cm$^{2}$), that is $\sim$ 45% of neutron converter atoms with respect to the EJ-420. Considering only the $^{6}$Li atoms, this proportion is further reduced to 15%. So, the neutron detection efficiency normalized to the $^{6}$Li content is much higher for the LiBO-based scintillator ($\sim$ 380% with respect to the EJ-420). However, taking into account that the cross-section of neutron capture on $^{10}$B is four times the one on $^{6}$Li, we expected to observe a higher counting rate. Nevertheless, the lower counts collected with LiBO detector as compared to EJ-420 can be explained by the fact that the alpha particles produced by the $^{10}$B capture reaction might lack sufficient kinetic energy to reach a ZnS:Ag nanocrystal and produce scintillation light. It is worth to note that a calculation performed with the LISE++ code^42^ revealed that the ranges of 1.5 MeV and 2.0 MeV alpha particles in the polydimethylsiloxane are around 7.3 and 10.6 $\upmu$m, whereas, the range of 2.7 MeV tritium ions is 60 $\upmu$m (for a better comprehension, an illustration of the range can be seen in Fig. 8). Besides, even if the light is produced, it could be easily self-absorbed. This statement is also supported by a previous work of our group, in which a scintillation material based on $^{6}$LiF nano-crystals, with a higher $^{6}$Li content (2.6 mg/$cm^2$) and without Boron^23^, showed a higher thermal neutron detection efficiency relative to EJ-420 (up to 90%).

In the next section, a Monte Carlo model of the ZnS:Ag/LiBO-based scintillator has been built in order to estimate the fraction of neutron capture events that actually produce a measurable signal.

### Computational model of the ZnS:Ag/LiBO-based scintillator

In order to estimate the fraction of neutron capture reactions (on both $^6$Li and $^{10}$B nuclei) that actually produce measurable electric signals, the experimental thermal neutron detection responses of three of the ZnS:Ag/LiBO-based scintillators, obtained with measurements performed using a $^{252}$Cf source, were compared with the ones predicted by a computational model, based on Monte Carlo simulations performed with the toolkit Geant4 v10.7^43^. The comparison is particularly focused on the scintillator samples having a weight ratio of 3:1 between the ZnS:Ag powder and the LiBO preparation. One of the scintillator samples with a weight ratio of 2:1 was studied, and no significant difference was observed with respect to the 3:1 samples.

In the code, the geometrical model of the LiBO-based scintillator consists of a disc of diameter 50 mm and thickness 0.4 mm (average of real samples). The composition of the disc is supposed to be homogeneous (all elements are distributed homogeneously). In the previous section was mentioned that the production of light is inherently dependent on the heterogeneity of the mixture, however, this Monte Carlo model is not intended to be used to simulate the yield and transport of scintillation light, in that case is necessary to consider the heterogeneity of the compound. The scope of the Monte Carlo model is to quantify the amount of neutron capture reactions, which, compared with the experimentally observed counting rate, can give an estimation of how many captures actually produced a measurable event.

Primary neutrons were emitted isotropically, with initial energy sampled from the well-known spontaneous fission neutron spectrum of $^{252}$Cf^44^. A total of $4 \times 10^7$ primary neutron events were simulated. The experimental set-up (detailed composition and geometry of the scintillators, polyethylene moderator, etc.) was reproduced in the G4VUserDetectorConstruction class. The neutron transport was managed with the high precision neutron model for low-energy neutrons (< 20 MeV) included on the QGSP_BERT_HP physics list. Using the G4Step and G4Track classes (implemented on the G4UserSteppingAction class), it was possible to track the history of each event during each step, and for example, have access to the position of the particle, time, kinetic energy, deposited energy, type of interaction, etc. To determine the total number of neutron capture reactions that took place either on $^6$Li or $^{10}$B, the energy deposited in the scintillator volume was registered when the reaction products (inside that volume) were an $\alpha$ particle or tritium in the case of $^6$Li, and an $\alpha$ particle or $^7$Li in the case of $^{10}$B. It is important to remark that this number should be equivalent to the maximum number of neutron-induced measurable signals produced by the scintillation detector when the experimental and simulated conditions are the same.

A comparison between the experimental and simulated total neutron efficiencies is presented in Table 1. The experimental total neutron efficiency was calculated by dividing the neutron count rate (events per second) by the number of neutrons emitted by the source in one second. The simulation results were computed in a similar way (i.e. total neutron capture reactions divided by the total primary neutrons emitted). For all scintillators investigated, the ratio between experimental and simulated efficiencies is roughly 10%, which means that only 10% of all neutron captures in the scintillator material result in a quantifiable event. The loss of these events could be explained by the combination of two factors: the alpha and tritium particles emitted after neutron capture do not interact with the ZnS:Ag compound, hence no scintillation light is emitted from the inorganic scintillator granules, and the opaque nature of the layer, that causes light scattering and, in turn, light loss along the path towards photomultiplier window.

**Table 1:** Experimental and simulated thermal neutron efficiencies registered by three of the ZnS:Ag/LiBO-based detectors, using the $^{252}$Cf source moderated with 6 cm of polyethylene.

ZnS:Ag/LiBO-based scintillator	Experimental efficiency (%), ± 0.0004	Simulated efficiency (%), ± 0.0004	Experimental to simulated eff. ratio (%), ± 0.05
LiBO 20% v/v	0.0133	0.1308	10.19
LiBO 30% v/v	0.0175	0.1622	10.76
LiBO 40% v/v	0.0201	0.1791	11.19

### Absolute light output of the ZnS:Ag/LiBO-based scintillator

**Figure 9:**
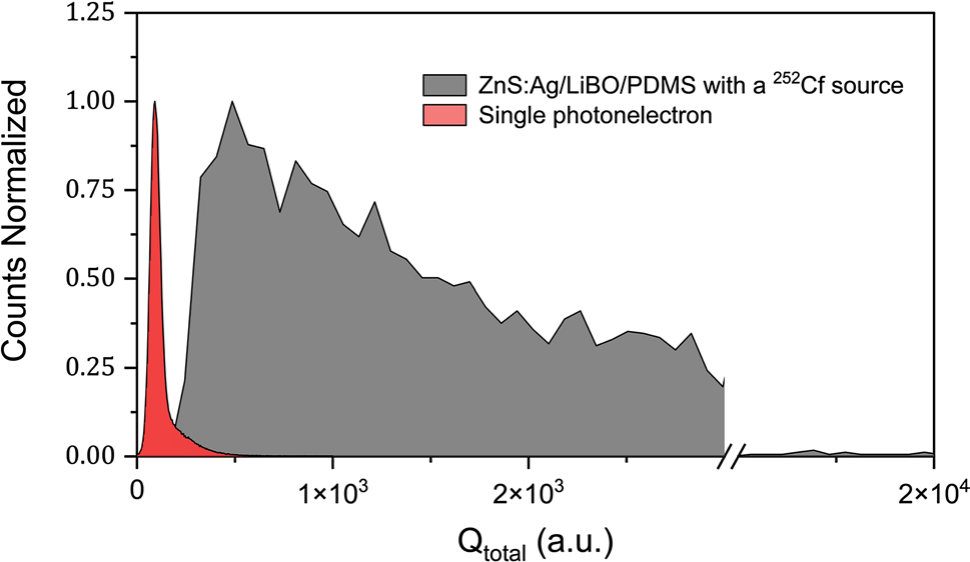
Pulse-height spectra of the single photoelectron response of the PMT, and of the ZnS:Ag/LiBO-based detector using a $^{252}$Cf source moderated with 6 cm of polyethylene.

Using the Bertolaccini method^45^, the average absolute light output of the ZnS:Ag/LiBO-based detector corresponding to one neutron capture event was determined. The Bertolaccini method is based on the comparison between the peak centroid position of the single photoelectron spectrum of the photomultiplier tube (PMT) and a determined position (full-energy peak, Compton edge, average energy, etc.) of the pulse-height spectrum of the scintillator corresponding to a specific experimental condition. In our case, a measurement performed with a $^{252}$Cf source (moderated with 6 cm of polyethylene) was used. Due to the fact that the pulse-height spectrum of the ZnS:Ag/LiBO-based scintillator is continuous, the position of the average $Q_{total}$ was used to obtain the average absolute light output. This was found to be (9000 ± 5%) ph/neutron-capture-event. However, the maximum value of light output was estimated to be around 30,000 ph/neutron-capture-event. An illustration of the pulse-height spectra comparison can be seen in Fig. 9. Our light output results were validated by a set of measurements performed using BGO and EJ-200 scintillators. The results obtained with these two standard scintillators are in agreement with previous studies^45,46^.

### Response of ZnS:Ag/LiBO-based scintillator under a high gamma rate field

The response of the ZnS:Ag/LiBO-based detectors under a high gamma field, when no neutron source is in the vicinity, was tested using five gamma sources ($^{241}$Am, $^{133}$Ba, $^{137}$Cs, $^{60}$Co and $^{22}$Na) with activities of about 300 kBq each one. The process involved taking a 12-h measurement with a set of gamma sources placed extremely close to the detector’s face. The number of fake counts identified in the thermal neutron region was selected in the 2D-PSD plot (same type of plot illustrated in Fig. 6c), and the counting rate achieved was 0.006 ± 5% cps, yielding a Gamma Rejection Ratio (GRR) parameter lower than 10$^{-11}$. Thus, a remarkable lack of sensitivity towards gamma-rays was demonstrated. This is a further characteristic that makes the ZnS:Ag/LiBO/PDMS detector a good candidate for neutron detection in a great variety of applications, as highlighted before. It is important to note that the EJ-420 exhibits higher gamma-ray sensitivity than the LiBO-based scintillator, with a GRR of 10$^{-7}$ (according to the manufacturer).

### ZnS:Ag/LiBO/PDMS scintillator coupled to a plastic scintillator: triple particle discrimination with a single hybrid assembly

**Figure 10:**
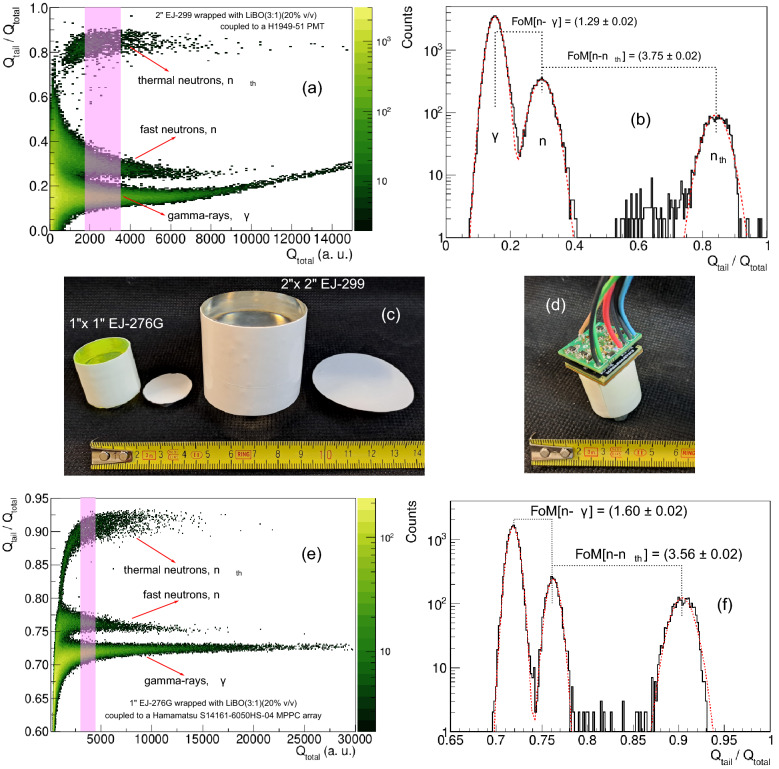
(**a**) 2D-PSD and (**b**) 1D-PSD plots of the ZnS:LiBO (3:1) (20% v/v)/PDMS-based detector covering the curved part and the top surface of EJ-299 plastic scintillator cylinder using a moderated $^{252}$Cf source. (**c**) Picture of the hybrid assemblies $1^{\prime \prime} \times 1^{\prime \prime}$ EJ-276G and $2^{\prime \prime} \times 2^{\prime \prime}$ EJ-299 wrapped with the LiBO-based scintillator. (**d**) Picture of the EJ-276G/LiBO hybrid detector coupled to a Hamamatsu S14161-6050HS-04 MPPC array. (**e**,**f**) (**e**) 2D-PSD and (**f**) 1D-PSD plots of the assembly using a moderated $^{252}$Cf source.

Coupling one of the ZnS:Ag/LiBO/PDMS scintillators (the ZnS:Ag/LiBO 3:1; 20% v/v was selected only because we had sufficient material to perform the tests) to a plastic scintillator, with fast neutron/gamma-ray discrimination capability, it is possible to build a highly efficient single detector unit able to discriminate between thermal neutrons, fast neutrons and $\gamma$-rays simultaneously. Two plastics scintillators were used for the tests, a 1$^{\prime \prime} \times 1^{\prime \prime}$ EJ-276G^46^ and a 2$^{\prime \prime} \times 2^{\prime \prime}$ EJ-299^47^. Figure 10c shows a picture in which both plastics are covered by the thermal neutron scintillator; two circular pieces used to cover one of the flat surfaces of the plastic scintillators are shown as well. The medium-sized EJ-299 plastic was coupled to the H1949-51 Hamamatsu PMT, whereas the small EJ-276G plastic, was coupled to an array of Silicon photomultipliers (Hamamatsu MPPC 4 $\times$ 4 array model S14161-6050HS-04), thus exploiting this recently tested device for n/$\gamma$ discrimination with organic scintillators, that proved to display remarkable benefits with respect to traditional assembly^48^ (see Fig. 10d).

In order to derive the main features of the ZnS:Ag/LiBO/PDMS scintillator, three types of assemblies were tested with the EJ-299 plastic scintillator. The first assembly consisted of covering one of the flat faces of the plastic with the ZnS:Ag/LiBO-based scintillator, while the other face was coupled to the PMT window. Then, in the second assembly, exploiting the flexibility of the ZnS:Ag/LiBO-based scintillator, the curved surface of the plastic scintillator was wrapped with the thermal neutron detector (see Fig. 10c). The third assembly was built for comparison purposes and consisted of the EJ-420 (disk of 2$^{\prime \prime}$ of diameter) optically coupled to one face of the EJ-299. A $^{252}$Cf source was placed in front of the detector, and it was moderated with 6 cm of polyethylene. The thermal neutron efficiencies, measured with respect to EJ-420, were (0.16 ± 2%) for the first experimental device and (0.28 ± 2%) for the second one. It means that full coverage of the curved walls of the 2$^{\prime \prime}$ plastic scintillator leads to a 75% enhancement in thermal neutron detection efficiency. In Fig. 10a,b the 2D-PSD and 1D-PSD plots corresponding to the measurement performed when the EJ-299 plastic scintillator is completely wrapped with ZnS:Ag/LiBO (3:1) (20% v/v)/PDMS scintillator are shown. In those figures, the cluster of $\gamma$-rays, fast and thermal neutrons induced events are clearly recognizable. The obtained Figures of Merit, taking into account the events in the shaded region in Fig. 10a, for the discrimination between fast neutrons and gamma-rays and fast neutrons and thermal neutrons are FoM[n-$\gamma$] = (1.29 ± 0.02) and FoM[n-n$_{th}$] = (3.75 ± 0.02) respectively. The light output of the events in the shaded region is approximately in the range 0.5–1 MeVee. Few visualized events are located between fast and thermal neutron zones and correspond to pile-up events. The $\gamma$-rays region in the 2D-PSD plot is slightly curved at high energies. This effect is probably caused by the non-linear behavior of the PMT, and can be minimized by operating the PMT at a lower bias or using another light converter device, such as silicon photomultipliers. In fact, in Fig. 10e,f the PSD plots of the EJ-276G/LiBO assembly coupled to the MPPC array are shown. As can be seen, excellent results in terms of particle discrimination and linearity are obtained. FoM values are reported in Fig. 10f and were obtained using the events contained in the shaded region in Fig. 10e (light output $\sim$ 0.4–0.6 MeVee).

For the assemblies using the two types of read-out devices (PMT and MPPC), the long G$_{L}$, and short G$_{S}$ integration gates were optimized until finding the largest FoM values for the discrimination between fast neutrons and the $\gamma$-rays. The discrimination between thermal neutron events and the other ones is easily achieved. In the case of the PMT (used with the EJ-299 scintillator) G$_{L}$ = 1.0 $\upmu$s and G$_{S}$ = 32 ns were obtained, while for the MPPC (EJ-276G scintillator) G$_{L}$ = 1.5 $\upmu$s and G$_{S}$ = 160 ns were used. In both cases, for events with light output larger than 0.4 MeVee, there is a complete separation between $\gamma$-rays and fast neutrons (FoM > 1.2), whereas, thermal neutron events are completely separated from the other events in any condition. This represents an excellent achievement, taking into account the results obtained in previous works when triple particle discrimination was explored, e.g. when plastic scintillators were loaded with lithium tetraborate nanoparticles^25^, good discrimination was found between $\gamma$-rays and fast neutrons, with a FoM value of $\sim$ 1.36, for events with a light output larger than 0.4 MeVee, but the thermal neutron events on the 2D-PSD plot were almost indistinguishable from the fast neutron events. Moreover, in another work performed by our group^30^, where an EJ-420 was coupled to an EJ-299 2$^{\prime \prime} \times 2^{\prime \prime}$, and the highest value of the FoM obtained between $\gamma$-rays and fast neutrons was around 1.1. Finally, Sharma et al.^31^ covered a 2$^{\prime \prime}$ EJ-299-33A plastic scintillator with a EJ-426 thin sheet and obtained good results in terms of triple particle discrimination, but taking into account the poor flexibility of the EJ-426 sheet, the assembly of this heterogeneous detector appears to be particularly tricky.

## Conclusions

A flexible and conformable thermal neutron scintillator has been produced by mixing a fully enriched Lithium Tetraborate preparation (LiBO) with a ZnS:Ag powder in a Polydimethylsiloxane matrix. The synthesis of the LiBO nanocrystals has been pursued following two different methods, the Pechini one, and the direct synthesis. In both cases, nanocrystals of LiBO were produced, although in the former method the tri-coordinated boron configuration is privileged over the tetrahedral one, as pointed out by X-ray diffraction and infrared analyses. For the production of the scintillators only one method was chosen and direct synthesis was preferred due to its higher yield. Six samples were produced varying the percentage of the ZnS:Ag/LiBO mix (20% v/v, 30% v/v, and 40% v/v) in the PDMS matrix, and varying the weight ratio between the ZnS:Ag powder and the LiBO preparation (3:1 and 2:1 respectively). Using a neutron flux generated at the Van de Graaff accelerator at Legnaro National Laboratories (Legnaro, Italy) and a $^{252}$Cf source, the neutron detection efficiency of the samples was assessed. Very good results were obtained compared to the performance of the commercial EJ-420 scintillator (from Eljen Technology), reaching up to 57% of relative thermal neutron detection efficiency, in particular with the ZnS:Ag/LiBO (2:1) (40% v/v)/PDMS scintillator. Moreover, in the near future, higher neutron converter concentration will be tested, in order to investigate the possibility to increase the neutron efficiency without compromising the current mechanical properties of the scintillator.

The average light output per neutron capture event of the scintillator was determined using the Bertolaccini method, and it was estimated to be (9000 ± 5%). On the other hand, the intrinsic response of the ZnS:Ag/LiBO-based detector to the presence of a $\gamma$-ray field was evaluated using a set of calibration gamma sources. It was found an excellent gamma rejection ratio parameter of GRR < $9\times 10^{-11}$. A bending test was done to highlight the flexibility capabilities of the LiBO-based scintillator. The proposed material outperformed the EJ-426 detector reaching a curvature radius as low as 1.5 mm. Furthermore, the response of the detector was modeled using the Monte Carlo method (Geant4 v10.7), and the comparison between the experimental and simulation results showed that only 10% of the neutron captures that occur in the scintillator material are able to produce a measurable event.

Finally, searching for the triple particle discrimination capability using a single hybrid unit, another configuration with the ZnS:Ag/LiBO-based detector was studied. The configuration takes advantage of the proposed detector flexibility, as two plastic scintillators (EJ-276G and EJ-299 from Eljen Technology) with fast neutron/gamma discrimination capabilities, were completely wrapped with the LiBO-based scintillator. Two different read-out devices were used, a standard PMT and a SiPM array (Hamamatsu MPPC). As a consequence, a compact detector capable of detecting and discriminating between $\gamma$-rays, fast, and thermal neutrons was obtained. Excellent discrimination was found between the three types of particles in all cases, with a FoM values larger than 1.2 for events with a light output greater than 0.4 MeVee. It is important to highlight the optimum results obtained using the MPPC as a light converter, taking into account that it is very compact, requires low power consumption, it exhibits high linearity, and it has an excellent particle discrimination performance. All the features of the ZnS:Ag/LiBO/PDMS detector studied and presented in this work make it a promising candidate to replace standard thermal neutron detectors, and, owing to its physical characteristics such as flexibility, conformability and lightness, to be employed in challenging scenarios where thin, flexible and cut to size panels are particularly useful and convenient.

## Methods

### Synthesis of ${^{6}}$Li$_2 ^{10}$B$_4$ O${_7}$ nanocrystals

The preparation of nanocrystals of LiBO has been already pursued based on the Pechini method to prepare a hybrid, polystyrene-based scintillator, featuring sensitivity to $\gamma$-rays and neutron, either in fast or slow^25^. The Pechini method is well known for the optimal size control and crystal quality achievable through it. In the first approach for the synthesis of LiBO, we adopted the following route: $^{6}$Li$_{2}$C$O_{3}$ and H$_{3}^{10}$B$O_{3}$ (Eurisotop, Sant Aubin, France; enrichment $^{10}$B 99%) in the stoichiometric ratio (1:4 in mol) have been mixed in water and polyvinylpyrrolidone (MW 4 $\times$ 10^4^) has been added (3:1 mol PVP:mol $^{6}$Li$_{2}$C$O_{3}$). Lithium carbonate was prepared from $^{6}$Li metal, present in the laboratory from previous experiments; it was dissolved with caution in HCl 2 M, and the solution was heated to dryness, producing pale yellow $^{6}$LiCl hydrated crystals. Then, a solution of (NH$_{4}$)$_{2}$C$O_{3}$ 2M (s = 250 g/l, 20 $^{\circ }$C) was added dropwise to 5 mL of $^{6}$LiCl 5M solution in stoichiometric amount to produce $^{6}$Li$_{2}$C$O_{3}$ (s = 12.9 g/l, 20 $^{\circ }$C). The low solubility of lithium carbonate was exploited to collect the crude product after centrifugation and washing with little water (yield 78%). The precursor’s solution ($^{6}$Li$_{2}$C$O_{3}$ and H$_{3}^{10}$B$O_{3}$) with PVP was stirred at 60 $^{\circ }$C for 1 hour, then it was poured in a large Petri dish and dried in an oven at 60 $^{\circ }$C overnight. The deep yellow, solid gel was pulverized in an agate mortar and transferred into an alumina boat. Calcination was carried out at 650 $^{\circ }$C for 2 h and produced a white powder. The yield was around 58%. Since both precursors are quite expensive, an alternative way of preparation of LiBO was pursued, aiming at maximizing the yield. The direct synthesis method was applied by weighing the proper amount of each reactant ($^{6}$Li$_{2}$C$O_{3}$: H$_{3}^{10}$B$O_{3}$ 1:4 in mol), mixing in the agate mortar and transferring it in the alumina boat for calcination. After 2 h at 650 $^{\circ }$C, an off-white, fluffy powder was recovered with a yield of 94% (Fig. 2).

### Analysis of ${^{6}}$Li$_2 ^{10}$B$_4$ O${_7}$ nanocrystals

Powders prepared through the Pechini method or direct thermal synthesis were analyzed for the crystal structure by high resolution-X-ray diffraction (HR-XRD, Panalytical, MRD) . The crystallite size was derived by applying the Scherer formula to the five most intense peaks of XRD pattern and the results are reported in Fig. 2b. FTIR spectroscopy was applied to precursors (lithium carbonate, boric acid) and to the final product obtained by the different routes in order to follow chemical structure changes and identify the type of coordination of boron in the resulting oxide. Composition and morphology were investigated by scanning electron microscopy-energy dispersive X-ray spectrometry (SEMEDS, Tescan).

### Laboratory tests

**Figure 11:**
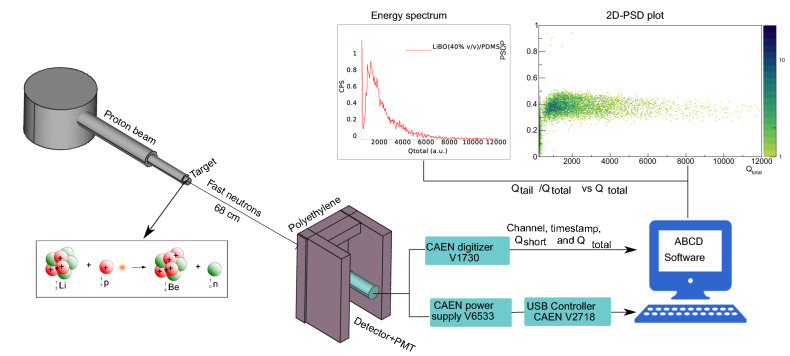
Schematic view of the experimental setup at the CN accelerator.

The performance of the six ZnS:Ag/LiBO-based scintillators was tested with a thermal neutron flux at the Van de Graaff accelerator facility at Legnaro National Laboratories, Legnaro-Italy. The neutron flux was obtained by impinging the proton beam from the accelerator into a $^{7}$Li target (700 $\upmu$ g/cm$^2$ thick), where the following nuclear reaction takes place $^{7}$Li(p,n)$^{7}$Be. The proton beam has a frequency of 3 MHz rep rate and a width of 2 ns for each pulse, the energy of the proton beam was set to 5.5 MeV, thus the energy of the fast neutron flux was 3.8 MeV. The detector was placed in front of the target at a distance of $\sim$ 68 cm and it was surrounded by a wall made of polyethylene blocks (with a thickness of around 6 cm, see in Fig. 11 the experimental set-up), to moderate the fast neutrons and obtain a thermal neutron flux.

The detectors were also tested using a $^{252}$Cf source (Activity of 2000 kBq and date 15/02/2014), which is a neutron-gamma emitter. The nucleus undergoes two types of disintegration: alpha decay (96.91%) and spontaneous fission (3.09%), which results in the emission of numerous neutrons and $\gamma$-rays. The fast neutrons emitted follow the so-called Watt energy spectrum (mean value around 2.1 MeV). A polyethylene moderator with a thickness of 6 cm was placed between the source and the detector. The distance between the detector and the $^{252}$Cf source was 15 cm. And for the tests with the hybrid detectors, 2 cm of polyethylene was used to boost the thermal neutron component of the flux.

### DAQ and data processing

Two photomultiplier tubes (PMT) were used to perform the measurements. A HAMAMATSU R6233 (operated at + 1250 V) was used for the absolute light output measurements and a HAMAMATSU H1949-51 (operated at − 1700 V) for the other tests. All scintillators were coupled optically to the PMTs using clear silicone grease. The signals were digitized by a fast CAEN digitizer model V1730, which has a sampling rate of 500 MSamples/s, and an ADC resolution of 14-bit. Modern digitizers are based on a programmable FPGA (Field Programmable Gate Array), where the digitized waveform can be pre-processed online. In this work the algorithm implemented in the FPGA provided for each triggered event: the timestamp, the total ($Q_{total}$), and the partial ($Q_{short}$) integrals of the waveforms. Also, the digitized waveforms can be recorded in order to perform an offline analysis of the data.

To supply the bias voltage to the photomultiplier, a CAEN power supply module, model V6533, was employed. The HV unit and the digitizer were controlled from the computer using a VME to PCI Optical Link Bridge (CAEN V2718 model) and a CAEN A3818 PCI Express CONET2 Controller (installed on a PC). The connections were made with an optical fiber using the CAEN CONET2 protocol. The data acquisition and the parameters of the readout electronics were managed using the ABCD (Acquisition and Broadcast of Collected Data) software^49,50^, released as an open-source project (https://github.com/ec-jrc/abcd), see in Fig. 11 the schematic view of the DAQ system.

A Figure of Merit (FoM) was used to assess the particle discrimination capabilities of the proposed assembly with the plastic scintillator.

2 $$\begin{aligned} FoM=\dfrac{\Delta }{\delta _{n}+\delta _{\gamma }}, \end{aligned}$$

where $\Delta$ and $(\delta _{n}+\delta _{\gamma })$, are obtained from a PSD parameter histogram (see Fig. 10b). $\Delta$ is the difference between the two mean values of the neutron and gamma distributions and $(\delta _{n}+\delta _{\gamma })$ is the sum of the $\gamma$-rays and neutron Full Width at Half Maximum (FWHM). The larger the FoM value, the better the system discrimination.

### Light output and gamma rejection parameters

The light output per neutron capture reaction of the ZnS:Ag/LiBO/PDMS detector can be calculated using the Bertolaccini method, where the position of the single photoelectron peak of the PMT was compared to the mean value of the detector’s pulse-height spectrum when the detector is exposed to a thermal neutron flux. The following expression is used in the calculation:

3 $$\begin{aligned} N_{ptm}(E)\propto N_{ph}(E) \cdot \eta _{L} \cdot QE(\lambda ) \cdot \epsilon , \end{aligned}$$

where $N_{ptm}(E)$ is the total number of photoelectrons collected at the anode of the PMT when a thermal neutron is detected, $\eta _{L}$ is the light collection efficiency, $N_{ph}(E)$ is the number of photons per energy unit produced in the scintillator at a given energy, $QE(\lambda )$ the quantum efficiency and $\epsilon$ the efficiency of photo-electron collection. The single photo-electron measurement can be taken with the scintillator coupled to the PMT, without any source in the vicinity.

Furthermore, another important parameter to be studied in the novel flexible and foldable composite detector is the Gamma Rejection Ratio (GRR), which is the intrinsic response of the neutron detector to the presence of a high $\gamma$-ray field when no neutron source is present.

4 $$\begin{aligned} GRR = \dfrac{N_{CPS}}{\Omega \cdot ( \sum _{i,j}^{} A_{i} \cdot \gamma _{i,j} ) }, \end{aligned}$$

where $N_{CPS}$ is the false neutron counts per second, $A_{i}$ is the activity of the i-th source, $\Omega$ is the solid angle and $\gamma _{i,j}$ is the intensity of the j-th gamma line of the i-th source. This characteristic of the detector was studied using five gamma sources ($^{241}$Am, $^{133}$Ba, $^{137}$Cs, $^{60}$Co, and $^{22}$Na), which were placed attached to the face of the detector.

## Supplementary Information

Supplementary Figure S1.

## Data Availability

The datasets generated and/or analyzed during the current study are available from the corresponding author on reasonable request.
