# Supplementary material for: Novel flexible and conformable composite neutron scintillator based on fully enriched lithium tetraborate
Source: Sci Rep. 2023 Mar 23;13:4799. doi: 10.1038/s41598-023-31675-9 (PMC10036633; doi:10.1038/s41598-023-31675-9)
Supplement: Supplementary file 1 — Supplementary Figure S1. [file 41598_2023_31675_MOESM1_ESM.docx]

Supplementary information

Fig. S1: SEM images of the composite ZnS:Ag/LiBO 3:1 (40% vol.). On the left, image taken with secondary electrons; on the right, image taken with backscattered electrons.

The dispersion of the composite in the case of ZnS:Ag/LiBO 3:1 40% vol. is shown, as derived from SEM analyses. On the right side, the same view field is acquired using backscattered electrons (BSE), so that higher average atomic number compounds produce brighter signal. It can be seen in the BSE image that ZnS:Ag grains display bright contrast, and have an average size of ~10 µm, in agreement with manufacturer claims, with irregular roundish shape. PDMS matrix is visible as dark grey areas, whereas LiBO particles are smaller than ZnS:Ag and are visible as grey areas laying on the bright grains, as evidenced in SE and BSE images by the red circles.
